# COVID-19 booster enhances IgG mediated viral neutralization by human milk *in vitro*

**DOI:** 10.3389/fnut.2024.1289413

**Published:** 2024-02-09

**Authors:** Vivian Valcarce, Lauren Stewart Stafford, Josef Neu, Leslie Parker, Valeria Vicuna, Tyler Cross, Olivia D'Agati, Sisse Diakite, Addison Haley, Jake Feigenbaum, Mahmoud Y. Al Mahmoud, Anjali Visvalingam, Nicole Cacho, Ivan Kosik, Jonathan W. Yewdell, Joseph Larkin

**Affiliations:** ^1^Department of Pediatrics, University of Florida, Gainesville, FL, United States; ^2^Division of Neonatology, Department of Pediatrics, University of Alabama at Birmingham, Birmingham, AL, United States; ^3^Department of Microbiology and Cell Science, University of Florida, Gainesville, FL, United States; ^4^College of Nursing, University of Florida, Gainesville, FL, United States; ^5^Department of Pediatrics, University of California Davis, Sacramento, CA, United States; ^6^Laboratory of Viral Diseases, National Institutes of Health, National Institute of Allergy and Infectious Diseases, Bethesda, MD, United States

**Keywords:** human milk, COVID-19, booster, antibodies, neutralization, IgG, stool

## Abstract

**Background:**

Facilitated by the inability to vaccinate, and an immature immune system, COVID-19 remains a leading cause of death among children. Vaccinated lactating mothers produce specific SARS-CoV-2 antibodies in their milk, capable of neutralizing the virus *in vitro*. Our objective for this study is to assess the effect of COVID-19 booster dose on SARS-CoV-2 antibody concentration and viral neutralization in milk, plasma, and infant stool.

**Methods:**

Thirty-nine mothers and 25 infants were enrolled from December 2020 to May 2022. Milk, maternal plasma, and infants' stool were collected at various time-points up to 12 months following mRNA COVID-19 vaccination. A subgroup of 14 mothers received a booster dose. SARS-CoV-2 antibody levels and their neutralization capacities were assessed.

**Results:**

Booster vaccination led to significantly higher IgG levels within human milk and breastfed infants' stool. *In vitro* neutralization of VSV-gfp-SARS-CoV-2-S-gp, a laboratory safe SARS-CoV-2 like pseudovirus, improved following the booster, with a 90% increase in plasma neutralization and a 60% increase in milk neutralization. We found that post-booster neutralization by human milk was highly correlated to SARS-CoV-2 IgG level. In support of our correlation result, Protein G column depletion of IgG in milk yielded a significant reduction in viral neutralization (*p* = 0.04).

**Discussion:**

The substantial increase in neutralizing IgG levels in milk and breastfed infants' stool post-booster, coupled with the decrease in milk neutralization capabilities upon IgG depletion, underscores the efficacy of booster doses in augmenting the immune response against SARS-CoV-2 in human milk.

## Introduction

Maternal vaccination during pregnancy and breastfeeding plays a crucial role in ensuring the health and protection of mothers and infants. Current guidelines from the Centers for Disease Control and Prevention recommend the whooping cough vaccine (Tdap), Influenza, Respiratory syncytial virus (RSV) and COVID-19 vaccinations for pregnant and/or lactating women (1). Extensive research has demonstrated the efficacy of maternal vaccination in safeguarding breastfeeding infants (2–5).

The initial two dose mRNA vaccination series has been shown to significantly enhance immunogenicity and elicit protection against COVID-19 infection in adults (6, 7) and children as young as 6 months old (8, 9). Halasa et al. (10) found that maternal vaccination during pregnancy was associated with lowered risk of COVID-19 hospitalizations in infants under 6 months. Though, our group and others show a waning of SARS-CoV-2 antibodies 6 months post vaccination completion (11–13), studies have now shown that the mRNA booster dose significantly reduces the incidence and severity of COVID-19 infections compared to unvaccinated or placebo-treated controls among the general population (14, 15). One study found that infants of mothers receiving a third mRNA dose during pregnancy had shorter hospital stays and decreased rates of hospitalizations compared to infants of unvaccinated and unboosted mothers (16).

The predominant antibody isotype in human milk is IgA, followed by IgG and IgM. IgA, particularly sIgA, plays an important role in pathogen neutralization in mucosa with broad binding activity (17). Although the placental transfer of IgG from pregnant mothers to the infants' systemic circulation is well established (18, 19), little is known about the human milk IgG function as it traffics to the infants' intestinal tract.

In our previous work, we have established the presence of SARS-CoV-2 IgA and IgG antibodies in human milk and breastfeeding infant stool following maternal mRNA COVID-19 vaccination during lactation (20, 21). Notably, we and others observed a significant increase in these antibodies after the initial two-dose series (22–26), with peak levels occurring 7 to 10 days after the second dose and a subsequent decline at 6 months post-vaccination (11). In this study, we aimed to analyze the SARS-CoV-2 antibody titers and *in-vitro* neutralization capability in human milk, maternal plasma, and infants' stool at 12 months post-initial vaccination series to investigate the potential booster effect.

## Methods

### Participants recruitment and study design

This prospective observational study was conducted at the University of Florida with institutional review board approval. The inclusion criteria comprised breastfeeding women aged 18 years and older who had either pre- or post-COVID-19 vaccination status and provided informed consent. Thirty-nine breastfeeding mothers and 25 infants were recruited at different timepoints between December 2020 and May 2022, either before or after receiving COVID-19 vaccination from Pfizer/BioNTech, Moderna, or Johnson & Johnson. Of those, 5 mother's and 1 infant's samples were not included in the analysis (3 mothers only participated at 1 time-point; and two participants received the J&J vaccine). Given significant differences in effectiveness and antibody response with J&J compared to mRNA vaccines, those two mother-infant dyads were excluded. Participants completed a questionnaire collecting maternal/infant demographics, medical and family history, and vaccination side effects upon agreeing to participate.

Maternal plasma, milk and infant stool samples were collected up to 7-time points relative to COVID-19 vaccination completion: pre-vaccination, 15–30 days after the first vaccine dose and then at 7–30 days, 60–75 days, 90–105 days, 6 and 12 months following 2-dose vaccination series completion (Supplementary Figure 1). Not all participants contributed samples at every listed collection time point. In our prior publications, we presented the results up to 6 months post-maternal vaccination. In this paper, data from 34 mothers and 24 infants was included with a focus on a subgroup analysis of longitudinal samples collected at 6- and 12-months post-vaccination series. We examined 9 paired milk samples and 13 paired maternal plasma samples to study the booster effect. These samples were collected at 6 and 12 months, with the booster shot administered in between.

### Sample collection and processing

Maternal blood samples were collected via venipuncture or finger prick in ethylenediaminetetraacetic acid-coated (EDTA) tubes at designated time points and centrifugated at 2000 × g for 10 min to separate plasma from cellular matter. Milk samples (10 mL−30 mL) were collected and stored at −20°C within 4 hours of collection. The samples were aliquoted and underwent centrifugation (500 × g for 15 min) to separate the aqueous layer, which was further centrifugated (3000 × g for 15 min) to obtain the final aqueous layer stored at −20°C. Stool samples were collected in diapers, refrigerated overnight or submitted on the same day, and stored at −80°C. The stool samples were diluted in sterile DPBS, vortexed, and centrifugated (1500 × g for 20 min) to obtain the supernatant. This supernatant was then placed in a clean tube, vortexed and centrifugated (10,000 × g for 10 min) to obtain the final supernatant, which was stored undiluted at −20°C.

### SARS-CoV-2 antibody measurement

Measurement of SARS-CoV-2-Specific IgA and IgG concentrations was performed using previously validated ELISA kits (20). Dilutions were made for plasma and milk samples, while infant stool samples were run undiluted. Negative controls and duplicate samples were included.

### *In vitro* neutralization

The neutralization capability of milk and plasma samples was assessed using SARS-CoV-2 spike glycoprotein-expressing vesicular stomatitis virus (VSV-gfp-SARS-CoV-2-S-gp) and infection competent BHK cells expressing the human ACE2 receptor in a fashion similar to those utilized in Stafford et al. with modification (11). In short, serially diluted milk (1:5, 1:20, 1:80, 1:320, 1:1280) or plasma (1:20, 1:100, 1:500, 1:2500, 1:12500) samples were incubated with VSV-gfp-SARS-CoV-2-S-gp for 1 h and after incubation this sample/virus mixture was added to BHK-ACE2 cells and incubated for 48 h. Following the 48 h, cell proliferation was measured using the MTT assay. The MTT assay is used to measure cellular metabolic activity as an indicator of cell viability. Metabolically active cells reduce tetrazolium salt [3-(4, 5-dimethylthiazol-2-yl)-2, 5-diphenyltetrazolium bromide or MTT] to purple formazan crystals (27, 28). Metabolically active cells have NAD(P)H-dependent oxidoreductase activity that assists in reducing MTT to formazan. The formazan crystals are then dissolved in DMSO and OD is read at 570 nm. OD values were normalized to a scale of 0%−100% infectivity using control values. Half maximal effective concentration (EC50), or the sample concentration in which 50% of cells are viable, values were calculated for each sample and used for quantitative analysis. Samples were run in duplicate.

### Plaque reduction assay

Plaque reduction assays, as described by Baer and Kehn-Hall with modifications were used to confirm our MTT neutralization results (29). Neutralization assays were initially set up as MTT assays, however after 48 h of infection, cells were fixed in 10% formalin for 1 h, followed by crystal violet staining for 15 min. Plates were then washed, imaged, and optical density was measured. EC50 values were calculated as stated above for quantitative analysis. Samples were run in duplicate.

### IgG depletion

IgG depletion was performed using NAb Spin Protein G columns (ThermoFisher) using manufacturer's instructions. Milk samples were diluted 1:2 in sterile DPBS and passed through Protein G columns to deplete samples of IgG. IgG-specific depletion was confirmed using SARS-CoV-2 IgG and IgA ELISA plates. Neutralization assays were then performed as described above in paired diluted milk samples and Protein G-depleted milk samples.

### Statistical analysis

Descriptive statistics characterized the demographics and clinical features of the study sample. Ordinary one-way ANOVA, Mann–Whitney *U-*tests, and paired *T*-tests were employed for comparisons, and Spearman correlation analysis was conducted to explore relationships between variables. Statistical test results were reported as p-values, and software such as SPSS and GraphPad Prism 9 were used for analyses and figure creation. An alpha threshold of < 5% (or *p* < 0.05) was used for declaring statistical significance. Geometric mean values with geometric mean SD were shown in the figures.

## Results

To assess how SARS-CoV-2 vaccination influences antibody composition, 39 lactating women and 25 infants were enrolled in the study. We analyzed the data from 34 mothers and 24 infants, with a focus on a subgroup analysis of longitudinal samples collected at 6- and 12-months post-vaccination series. We examined 9 paired milk samples and 13 paired maternal plasma samples to study the booster effect. These samples were collected at 6 and 12 months, with the booster shot administered in between. Pre-vaccination samples consisted of 25 milk and 16 plasma samples; 7–30 days post second dose samples included 24 milk and 23 plasma samples; 6-month samples included 15 milk and 18 plasma samples; 12 months included 11 milk and 15 plasma samples. Due to the small number of participants, samples from 60–75 days and 90–105 days timepoints were excluded from the analysis. 40 infant's stool samples were collected up to 12 months post-vaccination. The study population consisted primarily of White non-Hispanic women in their mid-30s and their infants with a median infant age of 10 months at enrollment (Table 1).

**Table 1:** Study participants characteristics^a^.

	***N* (%) or mean ±standards deviation**
**Maternal characteristics (*****n*** = **34)**	
Age (years)	34 ± 3.6
**Race**	
White	33 (97)
Asian	1 (3)
**Ethnicity**	
Non-Hispanic	23 (67)
Hispanic	6(18)
Not disclosed	3(9)
Body mass index (Kg/m^2^)	24.5 ± 3.9
History of allergies^c^	9 (26)
History of asthma^c^	4 (12)
History of inadequate immune response to vaccine^c^	2 (6)
Family history of cancer^c^	17 (50)
Family history of autoimmune disorder^c^	3 (9)
Antibiotic use in 6 months before enrollment^c^	10 (29)
Time postpartum at enrollment (months)	5.3 ± 5
**Vaccine brand (first 2 doses)** ^**c**^	
Moderna	13 (45)
Pfizer	20 (55)
**Booster brand (third dose)**	
Moderna	8 (57)
Pfizer	6 (43)
**Infant characteristics (*****n*** = **24)**	
Infant gender	
Female	10 (42)
Male	14 (58)
Infant age at the time of first stool collection (months)	4.8 ± 5.4
Infant age at 12 months stool sample collection (months)	18 ± 7

^*a*^Categorical data are given as the number of participants and, in parentheses, the percentage of the total. Continuous data are provided as means ± standard deviations.

^*c*^Missing data from 1 subject.

### Predominant SARS-CoV-2 IgG response in milk, plasma and breastfed infants' stool after mRNA COVID-19 booster

**Figure 1:**
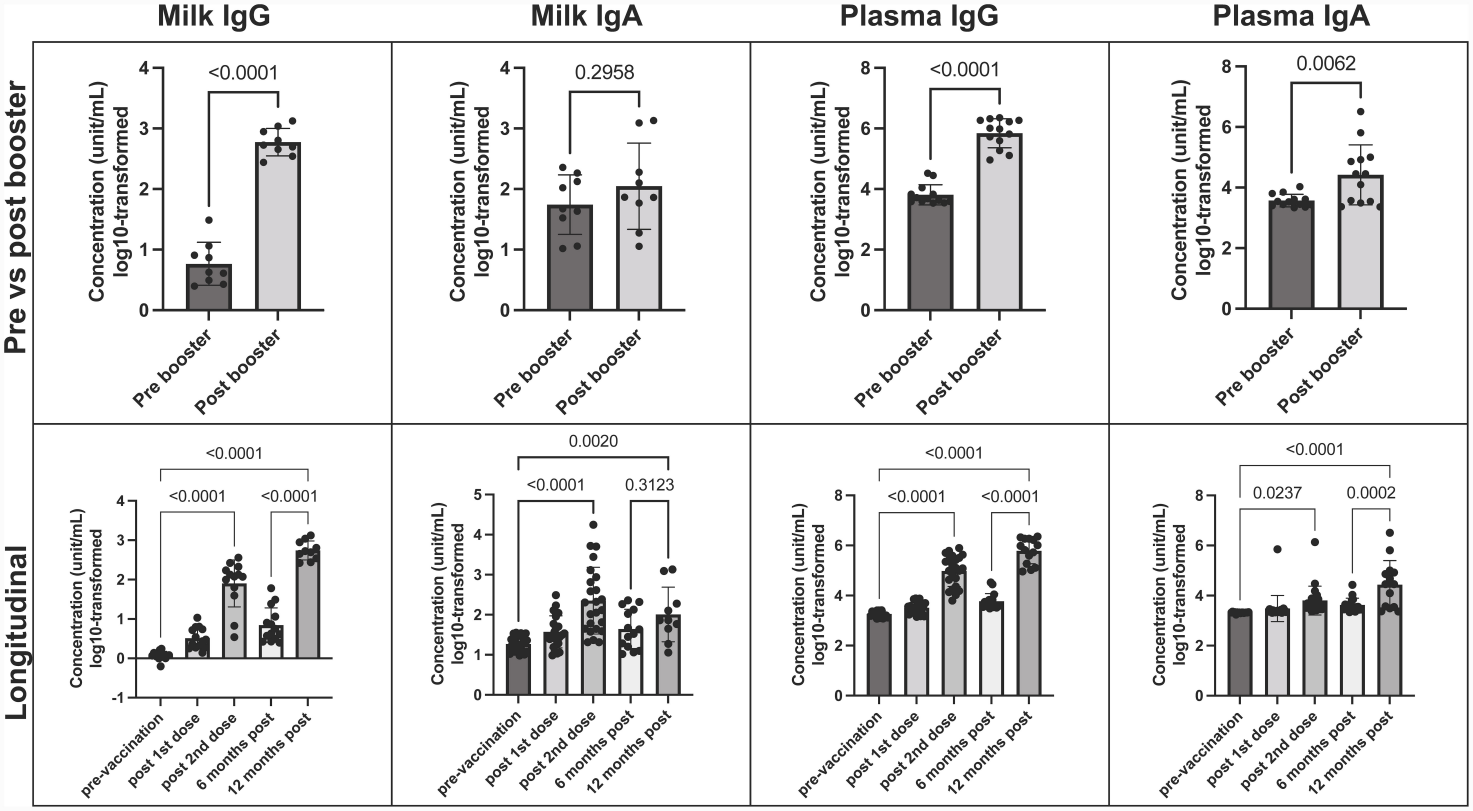
COVID-19 Booster promotes elevated SARS-CoV-2 specific IgG in human milk. Concentration of SARS-CoV-2 IgA and IgG in milk and plasma post booster dose **(top)**, and over 12-month vaccination course following initial COVID-19 vaccination **(bottom)**. Y axis = log10-transformed unit/mL; Graphs shown as geometric mean + SD. Pre vs. post booster statistical analysis = paired *T*-test; Longitudinal statistical analysis = Ordinary one-way ANOVA.

SARS-CoV-2 IgG and IgA concentrations were measured in maternal milk and plasma at 6 and 12 months post-initial vaccination series, with the booster administered in between. After the mRNA booster, there was a significant increase in SARS-CoV-2 IgG levels, evident in both human milk and plasma compared to pre-booster samples (*p* < 0.0001 in both) (Figure 1). Before the booster vaccination, SARS-CoV-2 IgG and IgA levels peaked between 7–30 days after the 2-dose vaccine series, with a predominant IgA response in milk. Although SARS-CoV-2 IgA did not show a significant increase after the booster, it did remain consistently above pre-vaccination levels, in which 9/11 (82%) milk and 12/15 (80%) plasma samples were above the positive cutoff. Throughout the 12-month period, we observed a less dynamic response in IgA levels in both human milk and plasma. The booster primarily triggered a rise in IgG levels, indicating a shift in the immune response toward a stronger IgG-mediated protection against SARS-CoV-2 (Figure 1).

Furthermore, a notable rise in SARS-CoV-2 IgG levels was evident in infant stool samples following the mother's booster dose, in comparison to pre-COVID negative controls. While we did not observe significant variations in antibody levels across specific time-points, the significance emerged when comparing the negative control group with all post-maternal vaccination samples, particularly those after the booster (*p* = 0.002) (Supplementary Figure 2).

### SARS-CoV-2 antibodies concentration positively correlate between milk and plasma

Using Spearman correlations, we found that SARS-CoV-2 antibodies in milk and plasma were positively correlated pre- and post-booster dose, where higher concentrations of milk IgG correlated with higher concentrations of plasma IgG (*p* = 0.007, R = 0.61 for IgA and *p* < 0.0001, R = 0.83 for IgG) (Supplementary Table 1).

Furthermore, we observed higher concentrations of SARS-CoV-2 IgG in milk and plasma in individuals who received the booster more recently, suggesting a time-dependent effect.

### Milk and plasma neutralization capabilities increase after the mRNA booster

In this study we wanted to robustly assess the potential of antibodies, present within the milk and plasma of booster vaccinated individuals, to neutralize SARS-CoV-2 viral activity *in vitro*. As such, we utilized two analysis methods to assess antibody-mediated inhibition of viral activity, using a laboratory-safe SARS-CoV-2-like pseudovirus. Whereas the MTT assay measured the ability of the pseudovirus to inhibit cellular metabolic activity, the plaque reduction assay was utilized to measure pseudovirus-mediated cell death (determined by changes in levels of intact, adherent cells).

**Figure 2:**
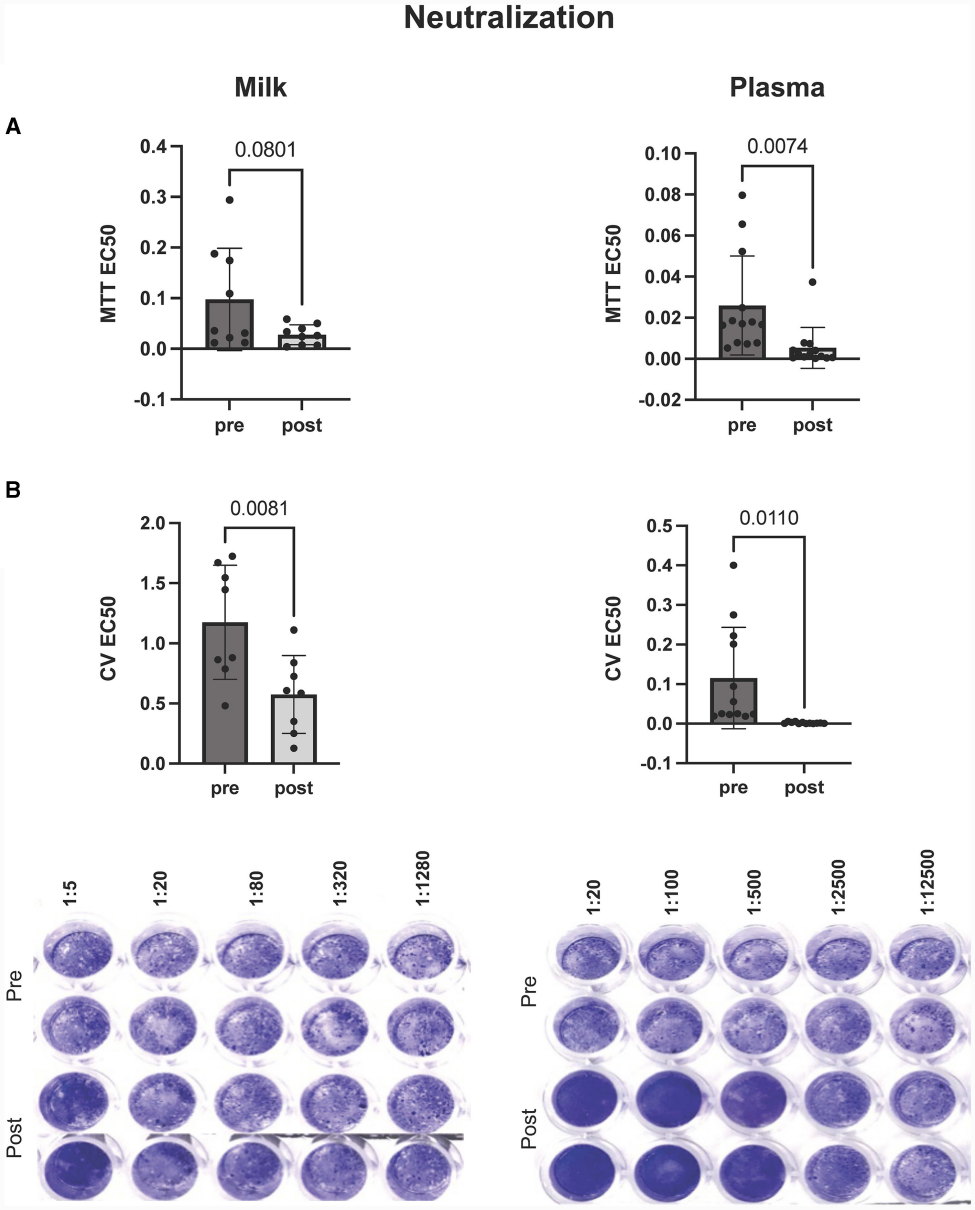
Inhibition of virus mediated cell death by milk (left) and plasma (right) pre and post mRNA COVID-19 booster dose. Neutralization of virus was assessed by **(A)** MTT and **(B)** plaque reduction assays. Y-axis = EC50 (half maximal effective concentration, sample concentration at which 50% of cells are protected from infection). Graph shown as geometric mean + SD; Statistical analysis = paired, *T*-test.

*In vitro* viral neutralization significantly increased in plasma by 90% after mRNA booster dose compared to pre-booster (*p* = 0.007), with a 60% increase in milk post booster dose (*p* = 0.08) (Figure 2A). These trends were confirmed using plaque reduction assays (Figure 2B). We found that plasma neutralization and plasma IgG concentration were significantly correlated (*p* = 0.0005, R = −0.64) pre and post mRNA booster dose (Supplementary Table 2). Milk neutralization and milk IgG and IgA concentrations were also correlated (*p* = 0.02 and 0.03, R = −0.56 and −0.52, respectively). These significant, negative correlations show the higher the SARS-CoV-2 IgG, the lower the EC50 value and higher neutralization.

Our results, from both the plaque reduction assay and MTT, show that cell survival and cellular activity is protected and preserved in cells treated with boosted plasma or milk during *in vitro* VSV-gfp-SARS-CoV-2-S-gp infection.

### Milk IgG depletion significantly decrease milk *in-vitro* neutralization capabilities

**Figure 3:**
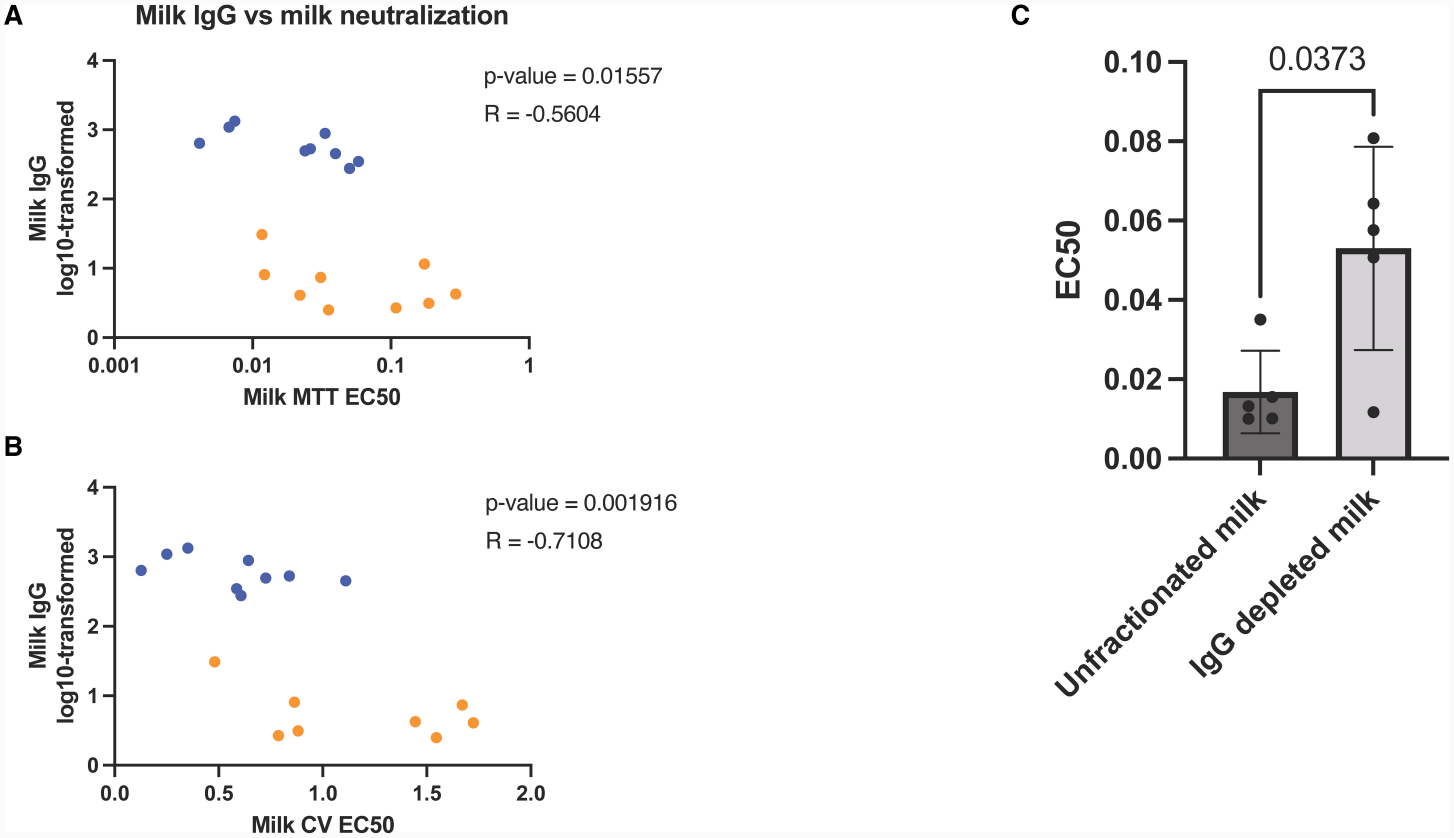
Human milk mediated viral neutralization is closely correlated to COVID-19 specific IgG post-booster. Spearman correlations of log10-transformed milk IgG vs milk neutralization assessed by MTT **(A)** and plaque reduction assay **(B)**. Orange dots represent pre booster dose values, blue dots represent post booster dose values. Graph showing neutralization capacity of milk before and after IgG depletion **(C)**. Y-axis = EC50 (half maximal effective concentration, sample concentration at which 50% of cells are protected from infection). Graph shown as geometric mean + SD; Statistical analysis = paired, *T*-test.

After observing the exponential increase in milk IgG concentration post-booster, along with its correlation with viral neutralization (Figures 3A, B), we aimed to investigate the role of IgG in *in vitro* viral neutralization. To accomplish this, we depleted IgG from milk samples using Protein G flow columns, which specifically bind human IgG. Subsequently, we measured viral neutralization using MTT assays. The results revealed a significant reduction in viral neutralization after the removal of IgG from the milk samples (*p* = 0.04) (Figure 3C).

To further validate our findings, we utilized SARS-CoV-2 IgG and IgA ELISAs, which confirmed that IgG concentrations were notably decreased through Protein G depletion, while IgA concentrations remained unaffected (Supplementary Figure 3).

## Discussion

A recent publication shows that COVID-19 remains a leading cause of death among children aged 0–19 from 2021 to 2022, with COVID-19 in infants under 1 year old, ranking as the seventh leading cause of death (30). A combination of emergent strains of SARS-CoV-2, an immature infant immune system, and a requirement for 6 months of age before vaccination, has prompted the CDC to recommend COVID-19 vaccination for pregnant and lactating mothers to combat infant risk during this critical life stage. Indeed, Halasa et al. (10) indicated that maternal vaccination with 2 doses of mRNA vaccine was associated with reduced risks of COVID-19 hospitalization, including critical illness among infants younger than 6 months of age. We and others have previously shown a significant increase in human milk derived SARS-CoV-2 IgA and IgG levels after initial mRNA COVID-19 vaccination series (20, 22–26). This increase was subsequently followed by a decline, noted 6 months post-vaccination (11, 31, 32). Notably, in this paper we show a significant increase in SARS-CoV-2 specific IgG within human milk post-booster immunization. Similar observations have been described by other researchers studying the humoral response to COVID-19 vaccines (32–35). IgG in milk was highly correlated to the increase in IgG present in the plasma post-booster. Notably, the ability of the milk to neutralize viral activity post-booster was highly IgG dependent, which drastically contrasted with pre-booster milk derived neutralization which was IgG independent (presumably IgA dependent).

Maternal immunity, primarily IgG, is transferred to the fetus during pregnancy via the placenta. Antenatal vaccination and/or infection infers pathogen-specific IgG transfer to the fetus, that wane over time (36). A recent study showed that the durability and quantity of placental derived IgG varied within infants as COVID-19 vaccination resulted in significantly higher transplacental persistence compared to infection after 6 months, with only 8% of infants born to infected mothers possessing antibodies at that time. The transplacental transfer of COVID-19 antibodies was also significantly lower at 6 months if the vaccination/infection occurred during the 3rd trimester compared to the second (37). Our study suggests that the consumption of human milk, from individuals receiving the COVID-19 booster, by infants may provide protection during the period of waning transplacental antibodies and the development of the infant's own immune system.

Although a critical role of human milk derived IgA, in the protection of postnatal infants has been well established, virus-specific IgG concentrations in milk have also been shown to be correlated with decreased risk for infant infection. Mazur et al. found that RSV Prefusion-F protein (pre-F) specific IgGs in human milk act as a clinical correlate for infant protection against respiratory syncytial virus illness. Particularly, pre-F IgG are lower in mothers' milk whose children became ill with RSV, compared to mothers of infant that did not develop RSV (38). Similarly, Fouda et al. found that milk-derived anti-HIV IgG has been shown to be correlated with lowered risk of mother-to-child transmission of HIV. Anti-HIV IgG in milk were positively correlated to plasma IgG, and IgG purified from both sample types had similar neutralizing capacity (39). These IgG in milk were shown to be correlated with neutralization and induction of antibody-dependent cellular cytotoxicity (36, 39).

Although there are lower levels of IgG compared to IgA in the gastrointestinal tract, recent studies have uncovered that among milk components, it is IgG, rather than IgA, that plays a crucial role in reducing pathogen loads and minimizing intestinal damage in breastfed pups following maternal infection or immunization in rodent models (40, 41). Caballero-Flores et al. (40) proved that maternal IgG coats, promotes phagocytosis and reduces pathogen attachment to intestinal mucosa in rodents pups (40). Another possible mechanism is that IgG binds to a broader microbial index (42). Indeed, Wang et al. (43) demonstrated that mRNA COVID-19 vaccination and/or SARS-CoV-2 infection significantly broadened the cross-reactivity of IgG in milk toward other human coronaviruses. In contrast to mice, evidence in humans has shown the passive diffusion of antibodies from the gut to the lamina propria is prevented by tight junction closure in infants born full-term (40 weeks) (44). Notably, however, it has been demonstrated, that the human neonatal Fc receptor (FcRn) was sufficient to promote transcytosis of IgG from the intestinal lumen to the lamina propria in a bidirectional manner using transgenic mice (45). Additional evidence suggesting a role of FcRn in modulating IgG mediated immunity at the lamina propria/intestinal lumen interface is the demonstration that FcRn receptors, present on the apical membrane of intestinal epithelial cells, can bind to IgG molecules found in the amniotic fluid following ingestion and facilitate endocytic transfer to the fetus (46, 47). Although it is possible that IgG may passively transfer from lumen to the lamina propria in extremely preterm infants (who may have a permeable gut), it is tempting to speculate that FcRn receptors may serve to actively transport maternal IgG derived from milk feeding into the systemic circulation of the infant.

We found a significant increase in SARS-CoV-2 IgG in the stool of infants receiving human milk post-vaccination. Interestingly, we previously found a statistically significant increase in SARS-CoV-2 specific IgG in infant stool up to 6 months post-maternal vaccination, even when the predominant human milk antibody isotype was IgA (11). Notably, ACE2 is highly expressed on the luminal surface of intestinal epithelial cells and SARS-CoV-2 is able to infect mature enterocytes within the intestine through expression of ACE2 (48, 49). Given the existing data, it is tempting to speculate that maternally derived SARS-CoV-2 specific IgG antibodies within the infant lumen may serve to inhibit viral binding to ACE2 receptors. Although future studies are necessary to fully elucidate the specific mechanisms whereby luminal IgG confers infant protection, these works highlight the protective role of maternal milk-derived IgG. Our current studies add to the existing body of literature, further underscoring the importance of human milk-derived IgA and IgG in promoting infant health.

## Limitations

This study highlights the role of human milk IgG and the transfer to breastfeeding infants after maternal mRNA vaccination. There are limitations to some of the results. First, there was a small sample size and limited diversity in this cohort of mothers. Although participants contributed data at various time points, no subjects contributed on all occasions. Furthermore, whether infants or mothers might have had a previously undiagnosed COVID infection, and how this may have interfered with results, cannot be known. We did not test the IgA secretory component, rather we assumed that IgA found in milk is majority secretory IgA. We did not explore other immune factors in milk (i.e., cell mediated immunity, cytokines, lactoferrin) that might be increased after maternal vaccination and contribute to milk neutralization capabilities. The mean age of our infant subjects at the time of enrollment was 5 months, and all were more than 6 months old by the 12 months stool collection time-point. Based on age, we assume that all of the enrolled infants had a mixed diet of solid food and human milk by 12 months collection time-point; this in combination with small sample size might have interfered with the stool antibodies concentration and neutralization capacity results, since exclusively breastfeeding infants may have higher antibodies levels.

Although we detect sIgA in stool, we cannot say for sure they are coming from milk rather than own infant intestinal mucosal production. However, several previously published works report that breastfeeding is positively associated with infant fecal sIgA concentration in the first 4 months of life, most notably when compared to sIgA in formula-fed fecal samples (50, 51).

## Data availability statement

The original contributions presented in the study are included in the article/Supplementary material, further inquiries can be directed to the corresponding author.

## Ethics statement

The studies involving humans were approved by the University of Florida Institutional Review Board 202003255. The studies were conducted in accordance with the local legislation and institutional requirements. Written informed consent for participation in this study was provided by the participants' legal guardians/next of kin.

## Author contributions

VVa: Conceptualization, Data curation, Funding acquisition, Investigation, Methodology, Project administration, Resources, Writing – original draft, Writing – review & editing. LS: Conceptualization, Data curation, Formal analysis, Investigation, Methodology, Resources, Software, Writing – original draft, Writing – review & editing. JN: Conceptualization, Funding acquisition, Project administration, Writing – review & editing. LP: Conceptualization, Methodology, Writing – review & editing. VVi: Investigation, Writing – review & editing. TC: Investigation, Writing – review & editing. OD'A: Investigation, Writing – review & editing. SD: Investigation, Writing – review & editing. AH: Investigation, Writing – review & editing. JF: Investigation, Writing – review & editing. MA: Investigation, Writing – review & editing. AV: Investigation, Writing – review & editing. NC: Conceptualization, Methodology, Writing – review & editing. IK: Methodology, Writing – review & editing. JY: Methodology, Writing – review & editing. JL: Conceptualization, Data curation, Funding acquisition, Investigation, Methodology, Resources, Supervision, Validation, Visualization, Writing – original draft, Writing – review & editing.
