# Supplementary material for: COVID-19 booster enhances IgG mediated viral neutralization by human milk in vitro
Source: Front Nutr. 2024 Feb 9;11:1289413. doi: 10.3389/fnut.2024.1289413 (PMC10884187; doi:10.3389/fnut.2024.1289413)
Supplement: Supplementary file 1 [file Data_Sheet_1.docx]

Supplemental Table 1.

| R  p-values | Milk IgA | Milk IgG | Plasma IgA | Plasma IgG | Infant stool IgA | Infant stool IgG | Time since last dose (days) |
| --- | --- | --- | --- | --- | --- | --- | --- |
| Milk IgA |  | 0.37  P = 0.13 | 0.61  P = 0.007 | 0.48  P = 0.04 | 0.13  P = 0.61 | 0.05  P = 0.84 | -0.31  P = 0.21 |
| Milk IgG | 0.37  P = 0.13 |  | 0.36  P = 0.14 | 0.83  P <0.0001 | 0.14  P = 0.59 | 0.28  P = 0.28 | -0.85  P <0.0001 |
| Plasma IgA | 0.61  P = 0.007 | 0.36  P = 0.14 |  | 0.68  P <0.001 | 0.24  P = 0.33 | 0.15  P = 0.56 | -0.39  P = 0.05 |
| Plasma IgG | 0.48  P = 0.04 | 0.83  P <0.0001 | 0.68  P <0.001 |  | 0.10  P = 0.68 | 0.30  P = 0.22 | -0.74  P <0.0001 |
| Infant stool IgA | 0.13  P = 0.61 | 0.14  P = 0.59 | 0.24  P = 0.33 | 0.10  P = 0.68 |  | 0.19  P = 0.46 | 0.17  P = 0.51 |
| Infant stool IgG | 0.05  P = 0.84 | 0.28  P = 0.28 | 0.15  P = 0.56 | 0.30  P = 0.22 | 0.19  P = 0.46 |  | -0.32  P = 0.19 |
| Time since last dose (days) | -0.31  P = 0.21 | -0.85  P <0.0001 | -0.39  P = 0.05 | -0.74  P <0.0001 | 0.17  P = 0.51 | -0.32  P = 0.19 |  |

Supplementary table 1. Non-parametric Spearman correlations of SARS-CoV-2 IgA and IgG in milk, plasma, and infant stool pre and post COVID-19 booster dose.

Supplemental Tables 2.

| R  p-values | Milk IgA | Milk IgG | Milk MTT | Milk CV |
| --- | --- | --- | --- | --- |
| Milk IgA |  | 0.37  P = 0.13 | -0.52  P = 0.03 | -0.42  P = 0.08 |
| Milk IgG | 0.37  P = 0.13 |  | -0.56  P = 0.02 | -0.69  P = 0.002 |
| Milk MTT | -0.52  P = 0.03 | -0.56  P = 0.02 |  | 0.54  P = 0.02 |
| Milk CV | -0.42  P = 0.08 | -0.69  P = 0.002 | 0.54  P = 0.02 |  |

| R  p-values | Plasma IgA | Plasma IgG | Plasma MTT | Plasma CV |
| --- | --- | --- | --- | --- |
| Plasma IgA |  | 0.68  P= 0.0001 | -0.47  P = 0.01 | -0.69  P <0.0001 |
| Plasma IgG | 0.68  P = 0.0001 |  | -0.64  P = 0.0005 | -0.88  P < 0.0001 |
| Plasma MTT | -0.47  P = 0.0147 | -0.64  P = 0.0005 |  | 0.78  P <0.0001 |
| Plasma CV | -0.69  P <0.0001 | -0.88  P = 0.0001 | 0.78  P <0.0001 |  |

Supplementary table 2. Non-parametric Spearman correlations of SARS-CoV-2 IgA and IgG in milk and plasma and neutralization capacity in milk and plasma. Milk or plasma MTT = MTT measured EC50 values, milk or plasma CV = plaque reduction assay (crystal violet) measured EC50 values.
