# Supplementary figures and images for: COVID-19 booster enhances IgG mediated viral neutralization by human milk in vitro
Source: Front Nutr. 2024 Feb 9;11:1289413. doi: 10.3389/fnut.2024.1289413 (PMC10884187; doi:10.3389/fnut.2024.1289413)

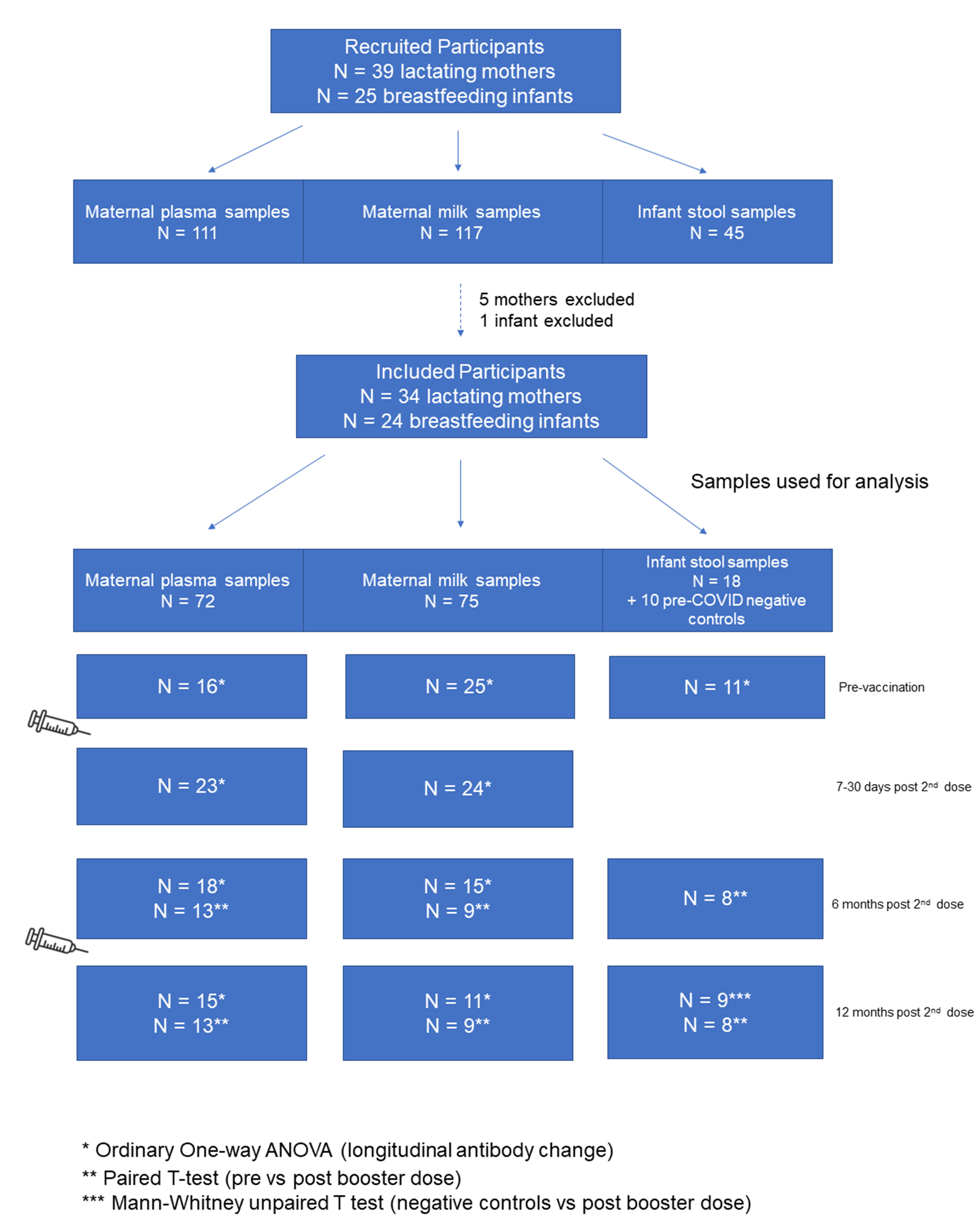

Supplement: Supplementary file 2 [file Image_1.TIF]
